# Differences in Primary Sites of Infection between Zoonotic and Human Tuberculosis: Results from a Worldwide Systematic Review

**DOI:** 10.1371/journal.pntd.0002399

**Published:** 2013-08-29

**Authors:** Salome Dürr, Borna Müller, Silvia Alonso, Jan Hattendorf, Cláudio J. M. Laisse, Paul D. van Helden, Jakob Zinsstag

**Affiliations:** 1 Veterinary Public Health Institute, Vetsuisse Faculty, University of Berne, Berne, Switzerland; 2 DST/NRF Centre of Excellence for Biomedical Tuberculosis Research, MRC Centre for Molecular and Cellular Biology, Division of Molecular Biology and Human Genetics, Faculty of Health Sciences, Stellenbosch University, Cape Town, South Africa; 3 Swiss Tropical and Public Health Institute, Basel, Switzerland; 4 University of Basel, Basel, Switzerland; 5 Royal Veterinary College, Hertfordshire, United Kingdom; 6 Division of Pathology, Veterinary Faculty, Eduardo Mondlane University, Maputo, Mozambique; Kwame Nkrumah University of Science and Technology (KNUST) School of Medical Sciences, Ghana

## Abstract

Tuberculosis (TB) is one of the most devastating infectious diseases worldwide. Whilst global burden estimates for *M. tuberculosis* infection (*Mt*TB) are well established, accurate data on the contribution of zoonotic TB (zTB) caused by *M. bovis* or *M. caprae* to human TB are scarce. The association of *M. bovis* infection with extrapulmonary tuberculosis has been suggested repeatedly, though there is little scientific evidence available to support this relationship. The present study aimed to determine globally the occurrence of extrapulmonary TB and the primary site (i.e. primary body location affected) of zTB in comparison with *Mt*TB, based on previously published reports. A systematic literature review was conducted in 32 different bibliographic databases, selecting reports on zTB written in English, French, German, Spanish or Portuguese. Data from 27 reports from Africa, America, Europe and the Western Pacific Region were extracted for analyses. Low income countries, in Africa and South-East Asia, were highly underrepresented in the dataset. The median proportion of extrapulmonary TB cases was significantly increased among zTB in comparison with data from registries of Europe and USA, reporting mainly *Mt*TB cases (47% versus 22% in Europe, 73% versus 30% in the USA). These findings were confirmed by analyses of eight studies reporting on the proportions of extrapulmonary TB in comparable populations of zTB and *Mt*TB cases (median 63% versus 22%). Also, disparities of primary sites of extrapulmonary TB between zTB and *Mt*TB were detected. Our findings, based on global data, confirm the widely suggested association between zTB and extrapulmonary disease. Different disability weights for zTB and *Mt*TB should be considered and we recommend separate burden estimates for the two diseases.

## Author Summary

Tuberculosis (TB) is one of the most devastating infectious diseases worldwide. The impact estimation of worldwide human TB is well established; however, that of TB transmitted by cattle, goat or sheep (i.e. zoonotic TB) is not. The affected body sites of human and zoonotic TB are repeatedly suggested to be different, which would influence the severity and impact of the diseases. The present study aimed to determine globally the association of affected body site and zoonotic TB by a systematic literature review. Data from 27 reports from Africa, America, Europe and the Western Pacific Region were included in the analyses. We found that the proportion of extrapulmonary TB was significantly higher in zoonotic TB than in human TB. Also, disparities of the specific body sites of extrapulmonary TB between zoonotic TB and human TB were detected. Our findings, which are based on global data, confirm the widely suggested association between zoonotic TB and extrapulmonary disease. Therefore, different measurements for estimating the impact of the two diseases should be considered.

## Introduction

Tuberculosis (TB) is a disease distributed worldwide, accounting for an estimated 1.5 million deaths annually [1]. The primary causative agent of human TB is *Mycobacterium tuberculosis*. Tuberculosis in cattle and goats is typically caused by *M. bovis* and *M. caprae*, respectively. Zoonotic transmission of these bacteria from livestock to humans is well documented [2]. Whilst *M. tuberculosis*-related human TB (*Mt*TB) is one of the most devastating infectious diseases worldwide, the public health relevance of zoonotic TB (zTB) appears to be minor in industrialized countries, where milk pasteurization is regularly performed [3]. However, in developing countries where control programmes for TB in livestock are absent and milk pasteurization is not done routinely, zTB may be of considerable importance [4]–[6]. A recent systematic literature review estimated incidence rates of zTB of approximately 7/100'000 human population/year in Africa [7]. This relatively low incidence rate notwithstanding, the study revealed that considerable rates of zTB exist in certain high-risk populations. Moreover, cases of zTB could be considerably under-diagnosed and consequently under-reported, particularly in developing countries [4]–[6].

Disease burden estimates are commonly calculated in terms of disability-adjusted life years (DALYs), taking into account disease frequency but also its severity and long-term effects (sequelae) [8]. The disability weights of zTB caused by *M. bovis* and *M. caprae* may differ from those of *Mt*TB. A main reason may be associated with the different primary sites (i.e. primary body location affected) of disease for zTB and *Mt*TB and potentially distinct secondary consequences resulting from this. An association of *M. bovis* infection with extrapulmonary TB has been suggested repeatedly in the literature, and current dogma suggests zTB to be transmitted to humans primarily by ingestion of contaminated products of animal origin rather than by aerosols, a hypothesis that would be in line with the more frequent extrapulmonary presentation of zTB in humans [3], [6], [9]. This view is mainly based on analysis of historical data from high-income countries and few other studies in geographically restricted regions or including small study populations. More comprehensive analyses investigating on a global scale the relative proportion of pulmonary versus extrapulmonary TB cases in humans and the primary sites of disease for zTB infection are missing. Other factors potentially affecting the clinical picture of zTB appear to be age, sex and co-morbidities (especially co-infection with the Human Immunodeficiency Virus; HIV). For example, an early study conducted in the UK revealed that TB caused by *M. bovis* in children was nearly always extrapulmonary [10]. Males may have a higher risk of infection with zTB due to their more frequent intensive contact to cattle, either at farms or in abattoirs [11].

The present study was mandated by the World Health Organization (WHO) Foodborne Disease Burden Epidemiology Reference Group. It aimed, based on available scientific evidence, to determine the proportion of extrapulmonary TB and the primary site of zTB and its association with patient demographic parameters and HIV co-infection status. It combines for the first time observational and intervention studies on this topic from all geographical regions and without restriction to specific study populations and used systematically selected high-quality data to explore the association between zTB and extrapulmonary TB.

## Methods

### Systematic selection of eligible reports and data extraction

**Figure 1:**
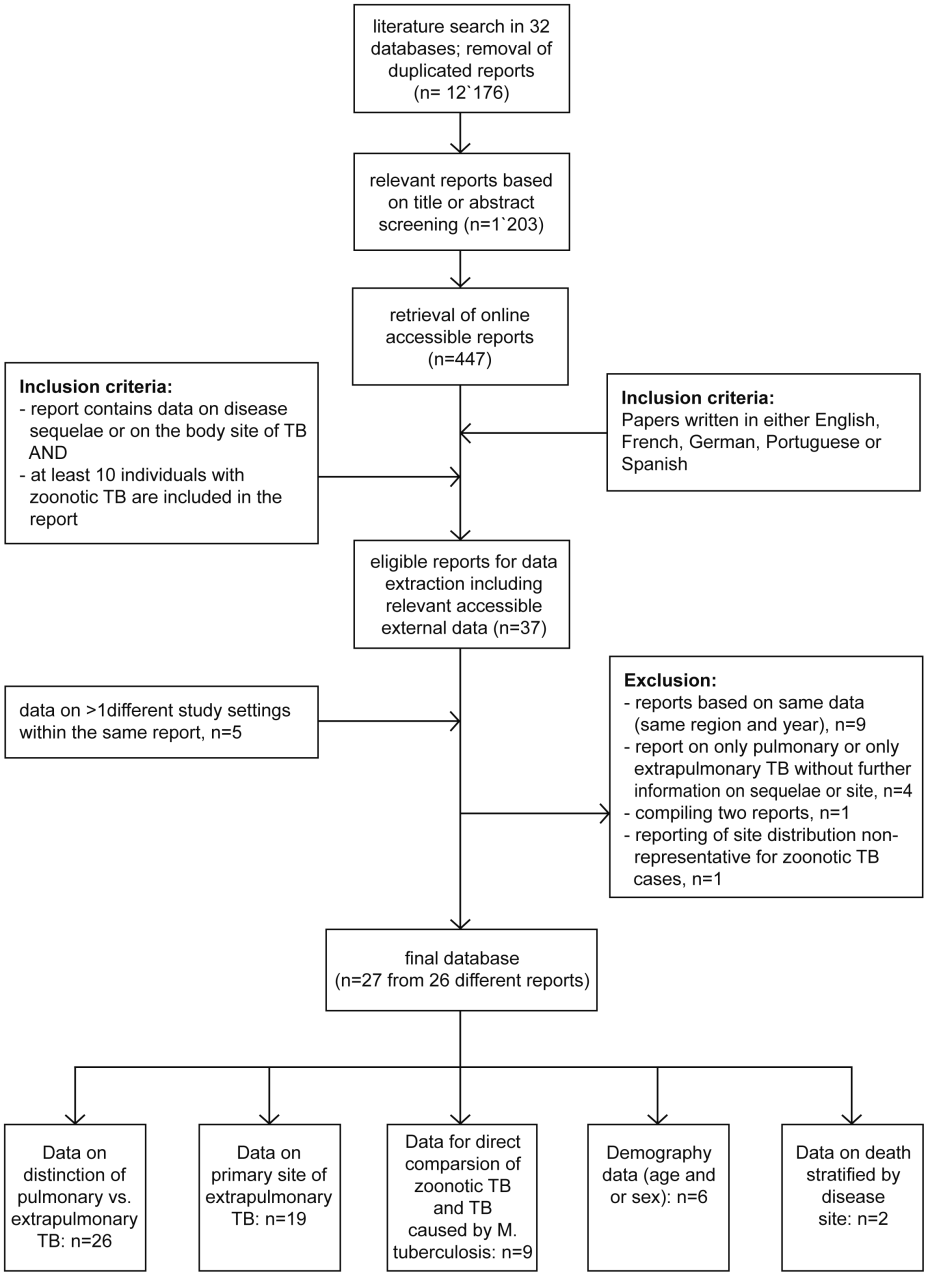
Selection procedure with inclusion and exclusion criteria for the 27 reports included in the study and their uses in analysis.

A systematic multi-lingual literature search was performed according to Cochrane guidelines with certain modifications (http://cochrane-handbook.org/). Thirty-two different bibliographic databases were searched for potentially relevant reports on putative zTB cases (*M. bovis* or *M. caprae* infections) in humans published until March 2010 using a highly sensitive search syntax (Tables S1 and S2). We included all types of observational and interventional studies on zTB or *M. bovis* and *M. caprae* infections in humans, unless the study reported exclusively on cases with evident human-human transmission. Before and after removal of duplicated reports 18'485 and 12'176 records, respectively, were identified (Figure 1). Titles and abstracts were screened to exclude reports which were unlikely to contain information on zTB cases; 1'203 potentially relevant reports remained of which 447 (37%) were available online and assessed for eligibility. We focused on reports available online for convenience while this was an important factor to improve the efficiency of the work. Moreover, it can be assumed that reports available online are of higher quality and that most of the more recent reports were available. Eligible records were written either in English, French, German, Spanish or Portuguese. Additionally, studies had to report on either the occurrence of extrapulmonary TB, primary site or sequelae of the disease. They had to include at least 10 individuals with zTB. No restrictions were made on the year when the study was undertaken. Eligibility of the relevant reports was assessed independently by three operators on 100 randomly selected reports. Ambiguities and diverging judgements were examined in order to harmonize the selection procedure. The remaining records were randomly assigned to one operator only. Thirty-seven reports were considered eligible for data extraction. Data was extracted stratified by WHO region, sex, age group and HIV co-infection of the patients, where possible. Data were sought for 20 variables (Table S3). After harmonizing the procedure of data extraction done by three operators based on 15 reports, the remaining reports were randomly allocated to one operator only. If any of the included reports were referring to relevant accessible external data which were not included in the database during earlier steps, these data were included as well in the analysis. Data on more than one study setting (different geographical regions and study periods) were available from two reports which enlarged the database by five records. A total of 15 reports had to be excluded for different reasons (Figure 1). The final database included 27 records from 26 different reports. Within these reports, differentiation of *M. bovis*, *M.caprae* and *M. tuberculosis* was done by molecular (e.g. PCR, spoligotyping), biochemical or both methods, or was not further specified for six reports. Anonymized human medical data was used.

### Source of *M. tuberculosis* data

Human TB registries from the USA and Europe were screened for information on the proportions of extrapulmonary TB cases and the primary sites of the disease. The reports did not distinguish between different species of the *M. tuberculosis* complex and consequently included *M. bovis* cases. Data from registries of the USA for the years 2005 to 2010 was used (Reported Tuberculosis in the United States, Department of Health and Human Services, [12]). For Europe, data were sourced from the EuroTB reports (InVS/KNCV, funded by the European Commission, [13]) and from the Tuberculosis surveillance in Europe reports (ECDC and WHO, [14]) for the years 1998 to 2006 and 2007 to 2010, respectively. The former included data from the European Economic Area (EEA) states, Israel and Switzerland; the latter included data of the EEA states only.

### Categorization of TB sites and analysis of the data

The primary sites of TB infection were categorized according to the ICD10 (International Statistical Classification of Diseases and Related Health Problems 10th Revision) online tool (http://apps.who.int/classifications/apps/icd/icd10online/). As an exception, we classified TB of the pleura as extrapulmonary, in agreement with most reports and registries that classify pleural TB as extrapulmonary TB, unless co-existing in a patient with pulmonary TB. Affected lymph nodes (also including non-defined lymph nodes) were counted as extrapulmonary TB whereas affected mediastinal lymph nodes were classified as pulmonary TB. Tuberculosis of the nervous system included meningeal TB, TB of the brain and spinal cord and of undefined nervous tissue. Tuberculosis of the intestines, peritoneum and mesenteric glands were categorized as intestinal TB. Patients with both pulmonary and extrapulmonary TB were defined as extrapulmonary cases throughout the study for both zTB and *Mt*TB.

We compared the frequency of extrapulmonary disease and of primary sites of zTB with *Mt*TB in two ways. First, zTB cases from the selected reports were compared with cases reported in TB registries (mainly *M. tuberculosis*), using the one sample Wilcoxon signed rank test. Second, information on comparable populations of *M. bovis* and *M. tuberculosis* cases within the same study were reported in eight reports regarding the occurrence of extrapulmonary TB and in four reports regarding the primary sites of TB infection (Table S4, footnote 3 and 4). The Wilcoxon matched pairs signed rank test was used for comparing the reported proportions. No weighting by study size was applied.

The bibliography database was stored in Reference Manager v11.0.1 bibliographic software. The data extracted was collated and stored in an access file. Data analysis was done using the statistical software R version 2.13.1 (http://cran.r-project.org/).

## Results

### Availability of data

**Figure 2:**
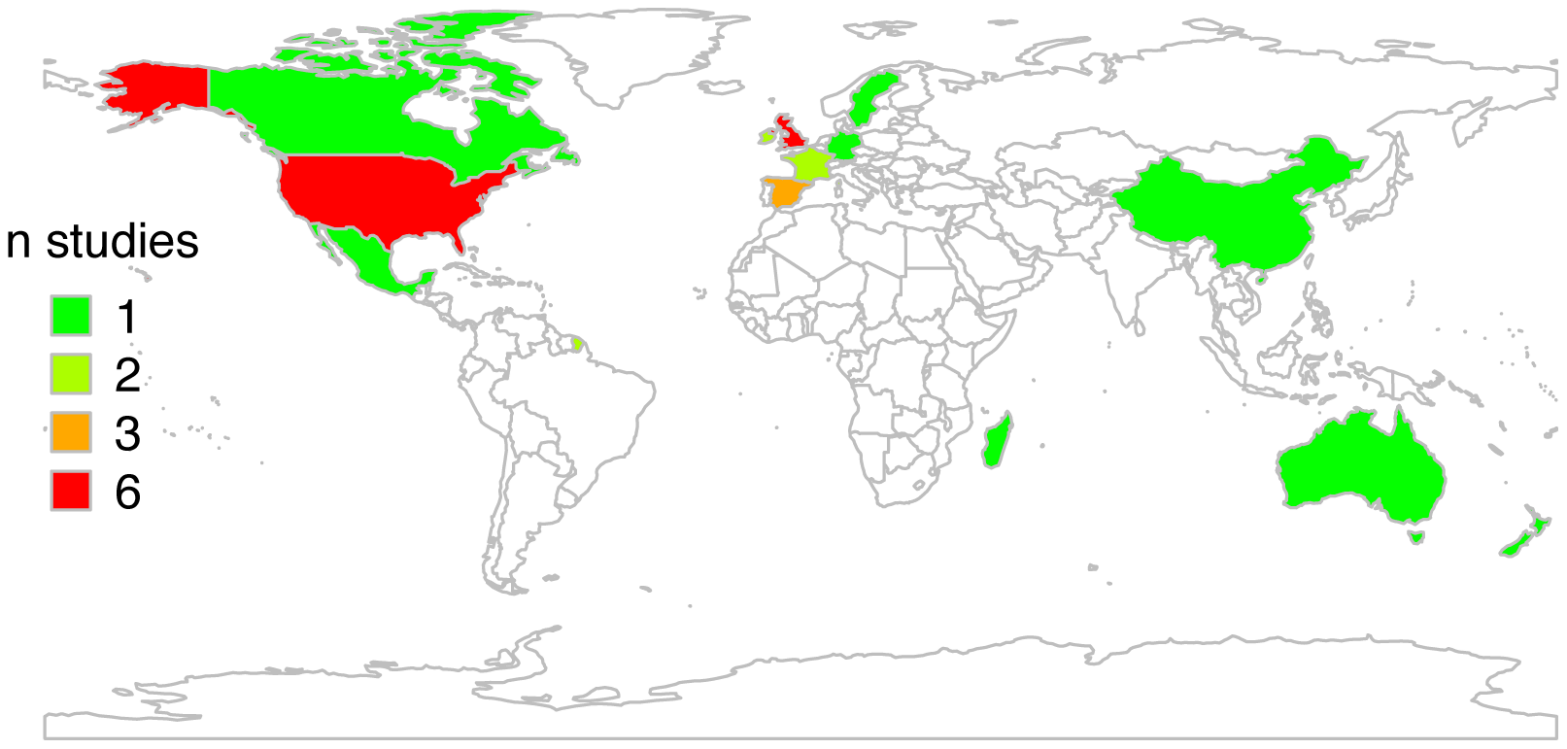
World map with the origin of data included in this study.

Twenty-seven reports from four different WHO regions (Africa, 1 report; America, 8; Europe, 15; Western Pacific region, 3) were included in the present study (Tables S4 and S5, Figure 2). The single study from Africa was done in Madagascar [15]. Half of the reports from Europe were carried out in the UK and Ireland. Six reports originated from the USA, 50% of which reported data from San Diego County, California, an area with a high proportion of Hispanic residents [16]–[18]. Reports included data from 1927–2007 whereas only 7 reports included data from earlier than 1980 (Table S4). Two studies from Europe reported on TB caused by *M. caprae* [19], [20] while the remaining of the zTB cases reported was caused by *M. bovis*. Sample sizes of zTB patients ranged from 10–296 cases with a median of 69 cases.

### Occurrence of extrapulmonary zTB and comparison to *Mt*TB

**Figure 3:**
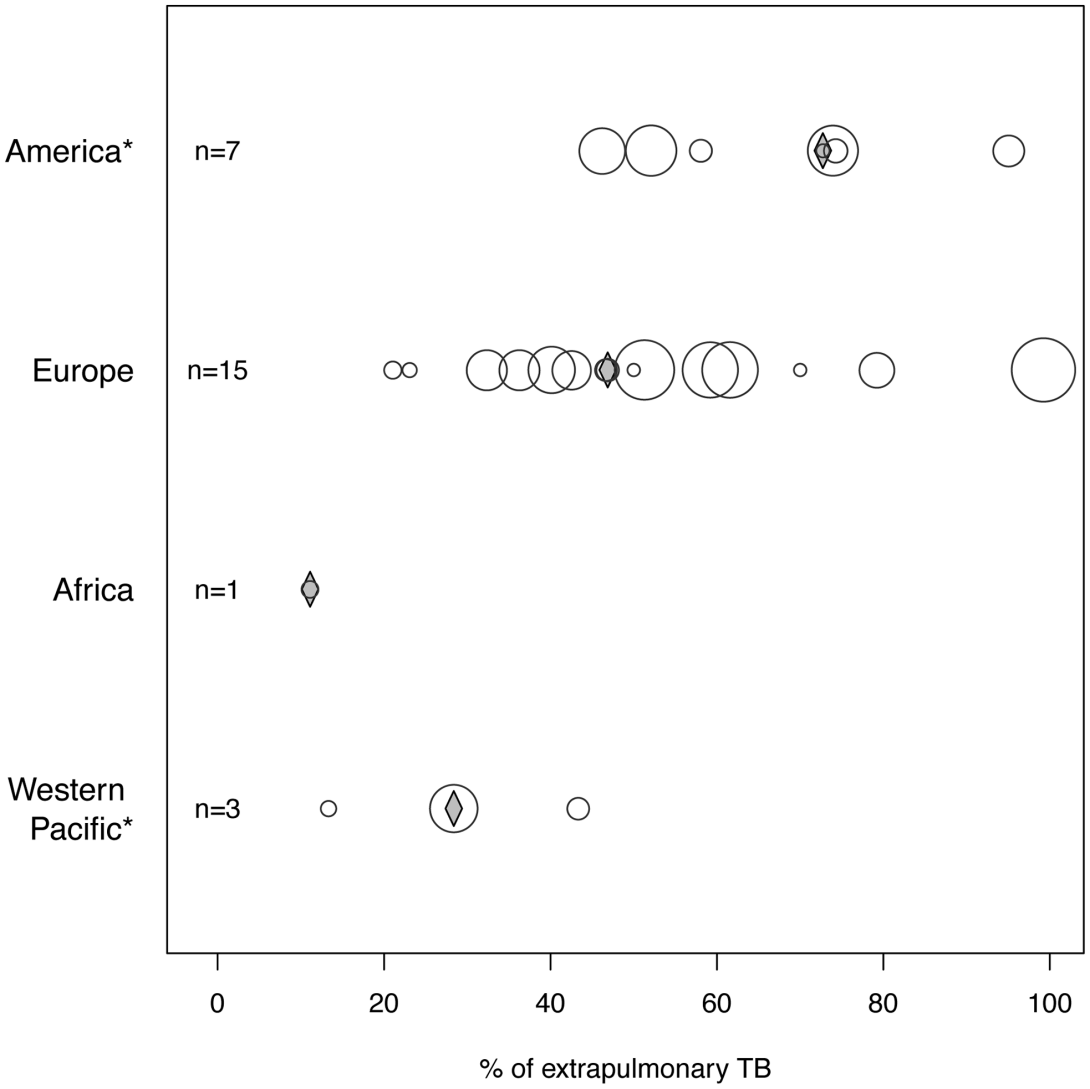
Proportion of extrapulmonary sites of TB per WHO region (n = the number of studies per region). The circle diameter is proportional to the number of patients included in the study. The diamond represents the median proportion of the respective region. *significant differences exists only between the WHO regions America and Western Pacific (two sample Wilcoxon signed rank test, n = 10, W = 21, p = 0.017).

Information on the proportion of extrapulmonary TB cases among all zTB patients was presented in 26 of the studies included (Table S4, footnote 1) and varied substantially between and within WHO region (Table 1, Figure 3). The region with the highest median value of extrapulmonary TB cases (73%, range 46–95%) was the Americas. Two studies from USA, which reported high proportions of extrapulmonary TB (95% and 74%, respectively), were conducted in specific populations. The first report included children only (0–15 years) [18] and the second was related to a food-borne outbreak caused by unpasteurized cheese, where a high proportions of extrapulmonary TB is expected [21]. Nevertheless, two other studies conducted in representative populations from the State of Michigan and the whole of the USA covering a period of 10 years, also reported proportions of 73% and 74% [22], [23]. According to the estimates in the studies conducted in Europe, the median proportion of extrapulmonary TB was 47% (21–99%). The only report from Africa presented a low proportion of extrapulmonary TB (11%) [15]. The three studies included from the Western Pacific region reported proportions of extrapulmonary TB from 13–43% with a median of 28% [11], [24], [25].

**Table 1:** Absolute numbers and proportions of pulmonary and extrapulmonary TB.

Report ID	Total number of patients	Pulmonary TB		extrapulmonary TB^a^		Unknown location
		n	*%*	n	*%*	n
**Africa**						
140	18	16	*89*	2	*11*	0
**America**						
485a	31	13	*42*	18	*58*	0
317	61	3	*5*	58	*95*	0
164	167	80	*48*	87	*52*	0
51	13	3	*27*	8	*73*	2
46	165	43	*26*	122	*74*	0
155	35	9	*26*	26	*74*	0
48	132	71	*54*	61	*46*	0
**Europe**						
598	264	2	*1*	262	*99*	0
338	38	17	*53*	15	*47*	6
485b	94	54	*57*	40	*43*	0
485c	102	65	*64*	37	*36*	0
612	77	16	*21*	61	*79*	0
588	201	82	*41*	119	*59*	0
149	13	10	*77*	3	*23*	0
425	30	16	*53*	14	*47*	0
515	10	5	*50*	5	*50*	0
521	228	78	*38*	125	*62*	25
162	19	15	*79*	4	*21*	0
74	296	113	*49*	119	*51*	64
219	166	85	*60*	57	*40*	24
87	11	3	*30*	7	*70*	1
13	110	69	*68*	33	*32*	8
**Western Pacific**						
337	148	106	*72*	42	*28*	0
134	34	17	*57*	13	*43*	4
55	15	13	*87*	2	*13*	0

The number of unknown location is presented for completeness and was subtracted from the total number of patients before calculating the proportions.

a includes some patients with both pulmonary and extrapulmonary TB.

The proportion of extrapulmonary zTB based on our dataset was significantly higher than within TB cases (mostly consisting of *Mt*TB) reported in TB registries. For Europe, the median proportion of extrapulmonary TB among cases of zTB was more than two-fold of that of *Mt*TB (47% for zTB vs. 22% for *Mt*TB, n = 15, V = 119, p<0.001). For the USA, this difference was even more pronounced (73% for zTB vs. 30% for *Mt*TB, n = 6, V = 21, p = 0.031).

**Figure 4:**
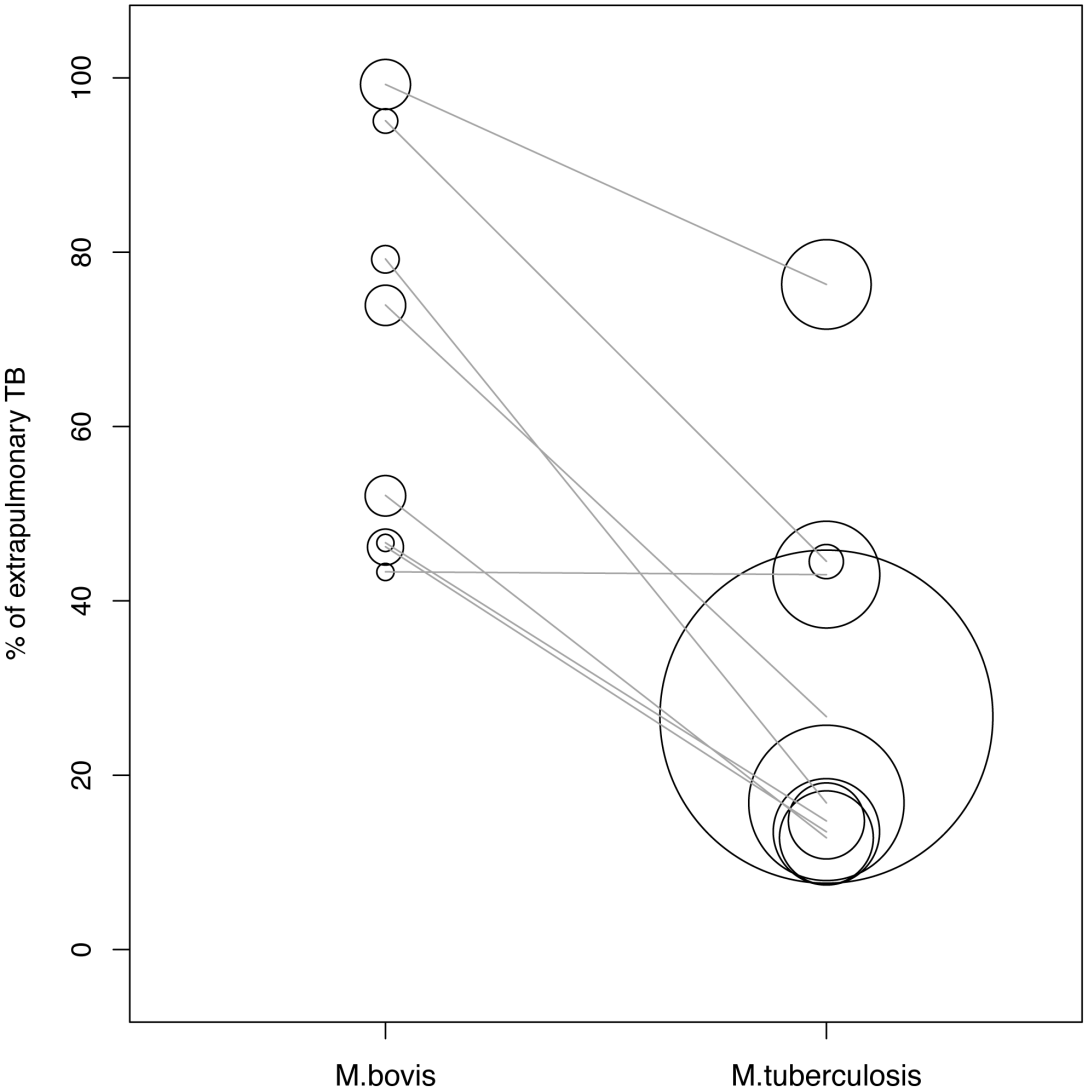
Proportion of extrapulmonary site of TB of the eight studies which reported on both, cases caused by *M. bovis* and *M. tuberculosis* (gray lines combine data from the same studies). The circle diameter is proportional to the number of patients included in the study.

These findings were confirmed by analyses of the proportions of extrapulmonary TB within the eight studies reporting on comparable populations of both, zTB cases and *Mt*TB cases (Table S4, footnote 3). The proportion of extrapulmonary TB was significantly higher among zTB cases than among *Mt*TB cases (median 63% vs. 22%, n = 16, V = 36, p = 0.008; Figure 4). Four of these reports originated from the USA [16]–[18], [22] with three from the San Diego County, California, two from England [26], [27] and one each from Ireland [28] and New Zealand [25].

Only one eligible study reported data stratified by sex and found a more than two-fold increased proportion of extrapulmonary cases in women (21/43 patients, i.e. 49%) than in men (20/103 patients, i.e. 19%) [11].

### Primary sites of extrapulmonary zTB and comparison to *Mt*TB cases

The primary sites of extrapulmonary zTB were reported and analysed in 19 reports (Table S4, footnote 2). Overall, lymph nodes (median of the proportions of affected lymph nodes among all extrapulmonary cases within each study = 30%) and the genitourinary system (median = 25%) were most often affected, followed by bones and joints (median = 13%), intestines and peritoneum (median = 6%) and the nervous system (median = 5%) (Table 2). This frequency distribution did not differ greatly between studies from Europe and the Americas with the exception of genitourinary TB having been detected in only two of five studies conducted in the Americas [29], [30] whereas in 8 of 10 studies conducted in Europe.

**Table 2:** Occurrence of specific sites of extrapulmonary TB of the 19 reports with available data.

Report ID	number of extra-pul-monary TB cases	TB of nervous system		TB of bones and joints		TB of genitourinary system		TB of skin and subcutaneous tissue		TB of intestines, peritoneum and mesenteric glands		TB of peripheral lymph-adenopathy		miliary TB		TB of the pleura		TB of other specified organs	
		n	*%*	n	*%*	n	*%*	n	*%*	n	*%*	n	*%*	n	*%*	n	*%*	n	*%*
**Africa**																			
140	2	0	*0*	1	*50*	0	*0*	0	*0*	0	*0*	1	*50*	0	*0*	0	*0*	0	*0*
**America**																			
485	18	0	*0*	1	*6*	12	*67*	0	*0*	1	*6*	4	*22*	0	*0*	0	*0*	0	*0*
317	58	3	*5*	5	*9*	0	*0*	0	*0*	12	*21*	31	*53*	5	*9*	0	*0*	2	*3*
51	8	1	*13*	0	*0*	0	*0*	1	*13*	1	*13*	5	*63*	0	*0*	0	*0*	0	*0*
46	79	7	*9*	6	*8*	0	*0*	0	*0*	14	*18*	45	*57*	0	*0*	0	*0*	7	*9*
39	17	1	*6*	0	*0*	6	*35*	0	*0*	0	*0*	10	*59*	0	*0*	0	*0*	0	*0*
155^a^	21	n.r.	*-*	n.r.	*-*	n.r.	*-*	n.r.	*-*	2	*10*	3	*14*	n.r.	*-*	n.r.	*-*	n.r.	*-*
**Europe**																			
598	262	8	*3*	93	*35*	4	*2*	107	*41*	0	*0*	50	*19*	0	*0*	0	*0*	0	*0*
338	14	0	*0*	6	*43*	3	*21*	0	*0*	0	*0*	5	*36*	0	*0*	0	*0*	0	*0*
485	40	4	*10*	9	*23*	12	*30*	0	*0*	3	*8*	12	*30*	0	*0*	0	*0*	0	*0*
485	37	2	*5*	4	*11*	25	*68*	0	*0*	0	*0*	6	*16*	0	*0*	0	*0*	0	*0*
588	119	8	*7*	22	*18*	45	*38*	1	*1*	8	*7*	32	*27*	0	*0*	0	*0*	3	*3*
149	3	0	*0*	1	*33*	0	*0*	1	*33*	0	*0*	0	*0*	1	*33*	0	*0*	0	*0*
425	14	1	*7*	0	*0*	4	*29*	0	*0*	2	*14*	4	*29*	0	*0*	0	*0*	3	*21*
515	5	0	*0*	0	*0*	0	*0*	0	*0*	1	*20*	2	*40*	0	*0*	2	*40*	0	*0*
521	125	6	*5*	19	*15*	50	*40*	3	*2*	4	*3*	41	*33*	2	*2*	0	*0*	0	*0*
87	7	1	*14*	1	*14*	1	*14*	0	*0*	1	*14*	3	*43*	0	*0*	0	*0*	0	*0*
**Western Pacific**																			
337	42	2	*5*	6	*14*	14	*33*	7	*17*	1	*2*	5	*12*	0	*0*	5	*12*	2	*5*
55	2	0	*0*	0	*0*	1	*50*	0	*0*	0	*0*	0	*0*	1	*50*	0	*0*	0	*0*

n = number of cases, % = proportion within the respective report.

a specific site of extrapulmonary TB was only recorded in 5 patients, this report was not included for calculation of the median proportion of primary sites; n.r. not reported.

Comparing the primary sites of extrapulmonary zTB of our dataset with the data from European TB registries reporting mainly *Mt*TB cases, extrapulmonary zTB was negatively associated with infections of the pleura (median = 0% vs. 27% in *Mt*TB, n = 10, V = 1, p = 0.004) and by trend with miliary TB (median = 0% vs. 6% in *Mt*TB, n = 10, V = 10, p = 0.07) but positively associated by trend with infections of the genitourinary system (median = 25% vs. 10% in *Mt*TB, n = 10, V = 46, p = 0.07) and bones and joints (median = 17% vs. 8% in *Mt*TB, n = 10, V = 46, p = 0.07). For the USA, no different lesion location was detected when comparing data from TB registries with those from zTB reports.

Information on the primary sites of extrapulmonary TB cases within comparable populations of zTB and *Mt*TB cases were reported in two reports from the USA [18], [22], and one each from Mexico [29] and England [26] of our dataset (Table S4, footnote 4). Lymph nodes (in all four reports), the genitourinary tract, the intestine tract and the skin tended to a positive association for extrapulmonary zTB (Figure S1). In contrast, bones and joints, the nervous system and the pleura were more often affected by trend in extrapulmonary *Mt*TB cases. No significant difference in primary sites between zTB and *Mt*TB was found using the Wilcoxon matched pairs signed rank test.

## Discussion

Global data on zTB infection and its relation to extrapulmonary TB are scarce. Studies investigating these aspects are needed in order to accurately estimate the global burden of this disease and to explore how it differs from the burden of *Mt*TB. Moreover, until now, the assumption of zTB being associated with extrapulmonary TB, has been based mostly on historical data from high-income countries and on few individual studies from geographically restricted regions or including small study populations. The present study analysed the frequency distribution of extrapulmonary zTB based on previously published reports. By combining the results from multiple reports without restriction to geographical regions or population groups, it aimed to explore the best possible evidence of the association between zTB and extrapulmonary TB. Additionally, the findings were compared to *Mt*TB using registries on human TB infections and comparing comparable populations of *M. bovis* and *M. tuberculosis* infected patients within the same studies. To our knowledge, this is the first study globally investigating the association between zTB and extrapulmonary TB as well as the primary sites of infection of extrapulmonary zTB.

Our study showed an increased proportion of extrapulmonary TB among zTB compared to *Mt*TB. While the comparison between zTB of the individual records and *Mt*TB of the registries may be questionable because of diverse study settings, the comparison between zTB and *Mt*TB within the same study is more robust and revealed an association of zTB and extrapulmonary TB as well. Similar results were reported previously, but mostly performed in geographically restricted regions (e.g. [9], [31]). The reason for this association may be explained by the different transmission routes, as zoonotic infection is primarily caused by consumption of unpasteurized dairy products. However, since the introduction of milk pasteurization in the 1950/60s in Europe and Northern America, patients with *M. bovis* infection in these regions suffered more often from reactivation of old infections, which mainly leads to pulmonary TB [3], [11], [30], [32]. This supports the decreasing trend of association of zoonotic and extrapulmonary TB over time that we observed in our analysis (Figure S2).

The occurrence of extrapulmonary zTB was very heterogeneous. This heterogeneity was found within and across different WHO regions and countries, likely due to different populations at risk in the different areas. However, there may be other factors underlying the different affected organs for zTB. The data extracted from our review does not allow a detailed investigation of these potential factors.

While we did not perform a formal risk of bias analysis, data were extracted from the reports to explore possible factors influencing our findings (Table S5). For example, the study population never corresponded to a representative population of a region. When the sampling strategy and the case finding strategy were reported, it was mostly and always, respectively, done by convenience and passively, whose effectiveness might differ between geographical regions and individual studies. Also, differentiation methods of *M. bovis* from *M. tuberculosis* were not homogenous between the different studies. However, we did not find a significant difference between the proportion of extrapulmonary TB in various reports which used molecular and biochemical identification methods (Wilkoxon rank sum test, n = 21, W = 64, p = 0.238). Furthermore, our analyses were influenced by the technical constraints of the studies included. For example, the paucibacillary nature of extrapulmonary TB complicates culture and speciation of bacteria, in particular, in low-income areas with limited laboratory infrastructure and still common use of the classical Löwenstein-Jensen medium which is less sensitive than the newer BACTEC systems. However, since this may affect all ecotypes of the *M. tuberculosis* complex, it is unlikely to affect our conclusions regarding the frequency and distinct primary sites of extrapulmonary infection for *M. bovis* and *M. tuberculosis*.

The proportion of extrapulmonary TB for zTB and *Mt*TB may be overestimated by the categorization of patients with both pulmonary and extrapulmonary TB as extrapulmonary cases. This categorization would be incorrect in cases where extrapulmonary disease is an advanced clinical picture of a primary pulmonary infection. However, as this categorization was applied for zTB and *Mt*TB, the conclusion for the comparison of the two agents may not be affected.

Comparison of the occurrence of primary extrapulmonary sites between zTB and *Mt*TB also revealed differences. This observation was made for studies conducted in Europe but not in the USA, which may be caused by different transmission routes in the two geographical regions. Further investigation would be needed to assess the transmission ways for different regions and populations at risk.

For Europe and North America, available data cover large regions over a period of more than 50 years (1954–2007) providing a good overview of the situation in these regions. Low income countries, as Africa and South-East Asia, were highly underrepresented in the dataset. As milk pasteurization in most of these countries is not yet routinely done [6], it is very likely that the consumption of dairy products is the main transmission route of zoonotic TB and therefore it is expected that the differences between the occurrence of extrapulmonary TB owing to zTB and *Mt*TB are more pronounced than in Europe and America. However, the almost complete lack of information from these areas makes it almost impossible to draw conclusion which are also valid for Africa and Asia. Additionally, our findings might be subject to publication bias because small studies without spectacular findings are less likely to get published.

Data on demographic parameters was rarely reported in the publications included in the review. Only one study, reporting on TB sites stratified by sex, had a sufficiently high sample size to draw any conclusion [11]. Also, data stratified by age category were limited with unevenly reported age classes and contradictory report results. Three studies reported higher proportions of extrapulmonary TB in young children [21], [33], [34]. Two of them were surveys which retrospectively analysed *M. bovis* infections of 10 and 11 hospitalized patients, respectively, over a period of 5 years from 1986–1990 in Spain and 6 years from 2000–2005 in France, respectively [33], [34]. It was hypothesized in both reports that the higher proportion of pulmonary TB was a result from reactivated older infection caused by dairy products, which had not been routinely pasteurized when these patients were born. Three patients from France, all aged between 35 and 40 years including two with extrapulmonary TB, were born in Africa where the risk of infection by unpasteurized milk products, and therefore extrapulmonary disease, is higher [33]. The third report was on a foodborne outbreak associated with unpasteurized cheese in New York City, where all of the five children below five years of age suffered from extrapulmonary TB while 9 of the 30 patients aged from 5–76 years showed pulmonary TB [21]. One study, a survey from 1995 in France, reported higher proportions of extrapulmonary TB in adults [35]. However, only two patients, both with pulmonary TB, were younger than 15 years. Interestingly, both of them were born in Africa from where extrapulmonary TB would be expected to be more likely. Finally one larger study including patients from 1986–1990 in England and Wales reported similar proportions of pulmonary and extrapulmonary TB for patients above and below 30 years of age [36]. Only two reports were available which reported on deaths among extrapulmonary and pulmonary zTB and they revealed contradictory results [16], [37]. To draw conclusions on the fatal outcome of extrapulmonary versus pulmonary zTB is therefore impossible. Only one report from Mexico included information on primary sites of extrapulmonary zTB stratified by the HIV co-infection of the patients [29] and it was therefore not possible to analyse the influence of HIV/AIDS on the TB sites.

For a proper DALY calculation, demographic and mortality data on the respective disease are required. The limitations of the availability of these data in our dataset impede therefore DALY calculations for zTB. However, the significant differences in the occurrence of extrapulmonary TB and primary sites of the disease between zTB and *Mt*TB detected in the present study demonstrate evidence for different disease sequelae. We may conclude that, regarding the differences in disease sequelae for the two causative agents, separate parameters for the DALY calculation should be used for zTB.

Our findings, based on previously published global data, corroborated the widely stated, albeit rarely demonstrated association between TB caused by *M. bovis* or *M. caprae* and extrapulmonary disease. Disparities between zTB and *Mt*TB were also suspected for specific sites of extrapulmonary TB. We do not know how well this conclusion fits the African and Asian situation, owing the almost complete lack of data. Therefore, investigations in these regions, where zoonotic TB is still relevant, are urgently required to assess the global universality of the conclusions. Nevertheless, there is evidence that the degree of disability weights for zTB and *Mt*TB differ and therefore, to measure most accurately the socio-economic impact of TB, burden estimation should be conducted separately for zTB and *Mt*TB.

## Supporting Information

Checklist S1 27-item check list of the Preferred Reporting Items for Systematic Reviews and Meta-Analyses (PRISMA) statement.
(DOC)

Figure S1 Proportion of occurrence of primary sites of extrapulmonary TB of the four studies which reported on both, cases caused by *M. bovis* and *M. tuberculosis* (gray lines combine data from the same studies). The circle diameter is proportional to the number of patients included in the study.
(TIF)

Figure S2 Proportion of extrapulmonary zoonotic TB over time of the study period whose start and end is represented by the lines (midpoint of the circles are midpoints of the study period). The circle diameter is proportional to the number of patients included in the study.
(TIF)

Flowchart S1 Four-phase Preferred Reporting Items for Systematic Reviews and Meta-Analyses (PRISMA) Flow Diagram; please consult Figure 1 for more detailed information.
(DOC)

Table S1 Searched bibliographic databases. Search syntax depended on whether or not the search engine did allow for the use of Boolean operators a complete or modified search syntax was used (Table S2). Total: Sum of records after removal of identified duplicates.
(DOC)

Table S2 Search syntax. Potentially relevant reports were identified using the search tools of the respective bibliographic databases (Table S1). At least one of the search terms under A had to be present in connection with at least one of the terms under B. The most sensitive search settings had been applied. If the respective search tool did not allow for the use of Boolean operators, all reports that were retrieved for any of the search terms under A were used. Search terms were translated into French for searches in French literature databases (Table S1).
(DOC)

Table S3 List of variables for data extraction of the eligible reports.
(DOC)

Table S4 Information on the included 27 records stratified by WHO region.
(DOC)

Table S5 Data extracted from the included 27 reports.
(XLS)
