# Supplementary figures and images for: Differences in Primary Sites of Infection between Zoonotic and Human Tuberculosis: Results from a Worldwide Systematic Review
Source: PLoS Negl Trop Dis. 2013 Aug 29;7(8):e2399. doi: 10.1371/journal.pntd.0002399 (PMC3757065; doi:10.1371/journal.pntd.0002399)

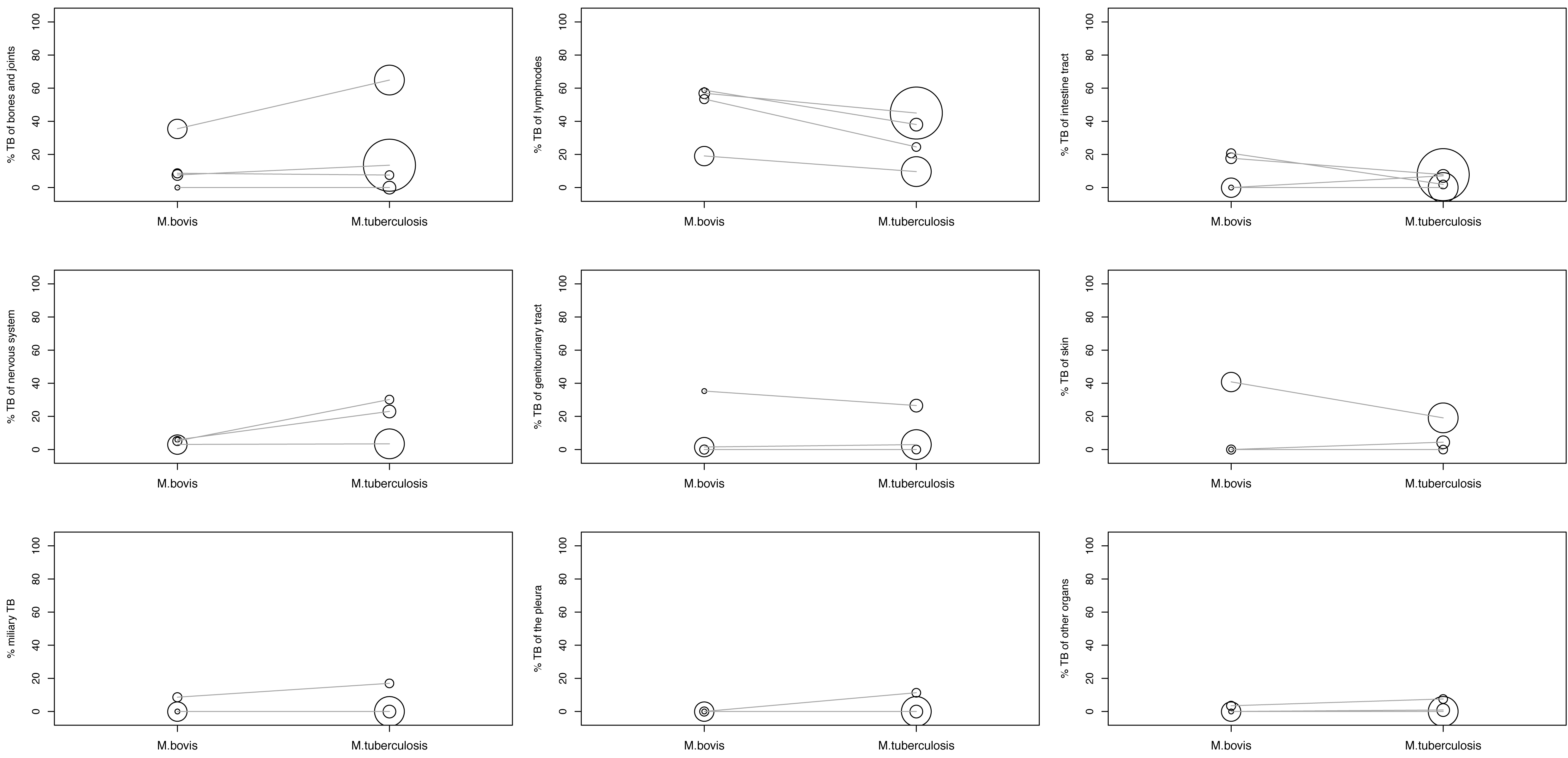

Supplement: Figure S1 — Proportion of occurrence of primary sites of extrapulmonary TB of the four studies which reported on both, cases caused by M. bovis and M. tuberculosis (gray lines combine data from the same studies). The circle diameter is proportional to the number of patients included in the study. (TIF) [file pntd.0002399.s002.tif]

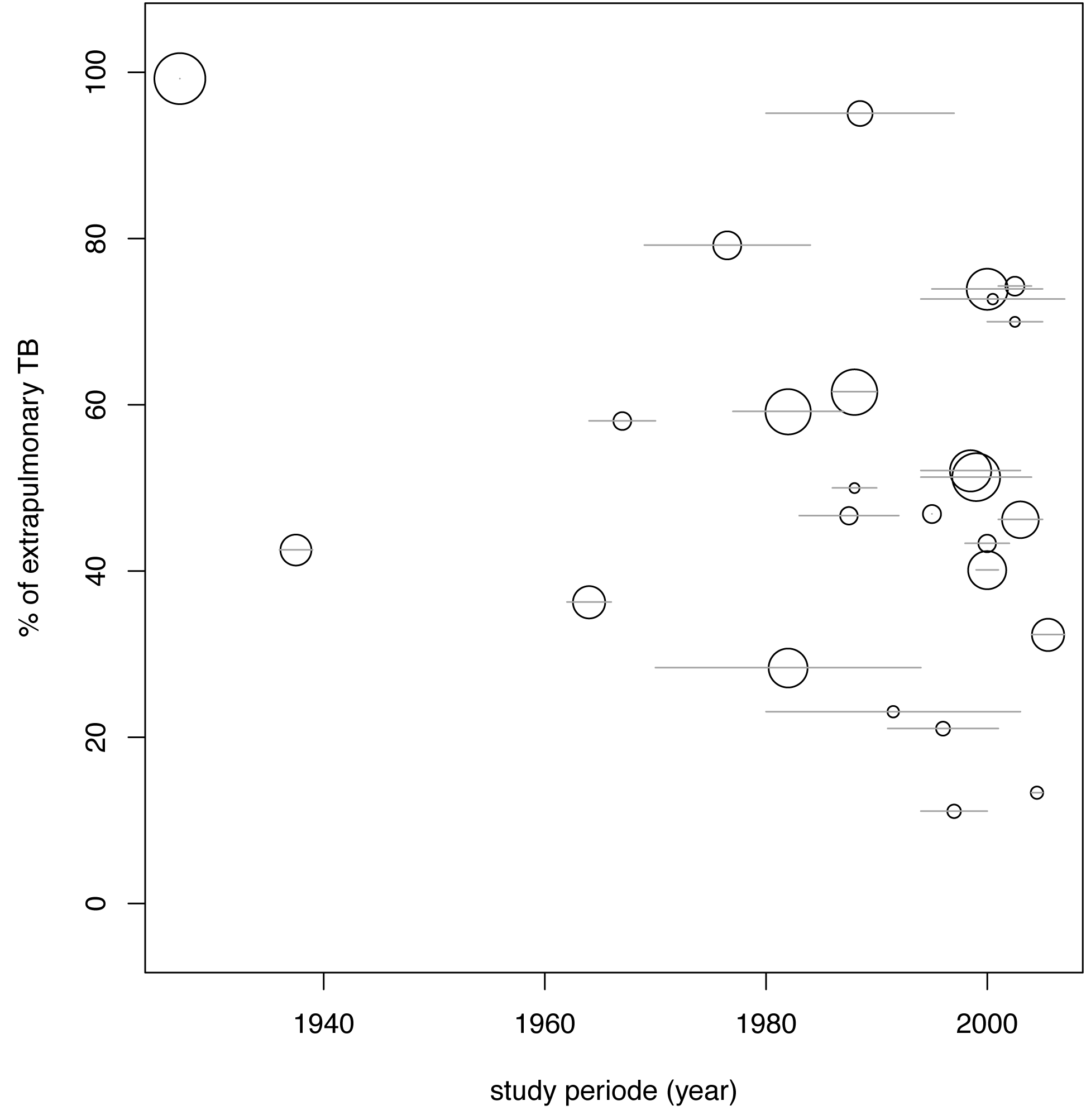

Supplement: Figure S2 — Proportion of extrapulmonary zoonotic TB over time of the study period whose start and end is represented by the lines (midpoint of the circles are midpoints of the study period). The circle diameter is proportional to the number of patients included in the study. (TIF) [file pntd.0002399.s003.tif]
