# Supplementary material for: Differences in Primary Sites of Infection between Zoonotic and Human Tuberculosis: Results from a Worldwide Systematic Review
Source: PLoS Negl Trop Dis. 2013 Aug 29;7(8):e2399. doi: 10.1371/journal.pntd.0002399 (PMC3757065; doi:10.1371/journal.pntd.0002399)
Supplement: Flowchart S1 — Four-phase Preferred Reporting Items for Systematic Reviews and Meta-Analyses (PRISMA) Flow Diagram; please consult Figure 1 for more detailed information. (DOC) [file pntd.0002399.s004.doc]

**Flowchart S1:**

**
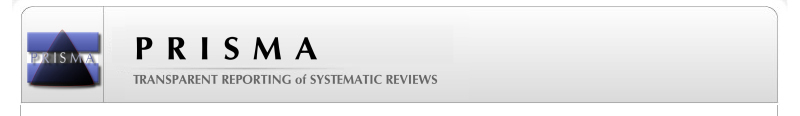
Four-phase Preferred Reporting Items for Systematic Reviews and Meta-Analyses (PRISMA) Flow Diagram; please consult Figure 1 for more detailed information**

**Screening**

**Included**

**Eligibility**

**Identification**

Records identified through database searching
(n = 18485 )

Additional records identified through other sources
(n = 0 )

Records after duplicates removed
(n = 12176 )

Records screened
(n = 12176 )

Records excluded
(n = 11729 )

Full-text articles assessed for eligibility
(n = 447 )

Full-text articles excluded, with reasons
(n = 420 )

Studies included in qualitative synthesis
(n = 27 )

Studies included in quantitative synthesis (meta-analysis)
(n = NA )
