# Supplementary material for: Differences in Primary Sites of Infection between Zoonotic and Human Tuberculosis: Results from a Worldwide Systematic Review
Source: PLoS Negl Trop Dis. 2013 Aug 29;7(8):e2399. doi: 10.1371/journal.pntd.0002399 (PMC3757065; doi:10.1371/journal.pntd.0002399)
Supplement: Table S1 — Searched bibliographic databases. Search syntax depended on whether or not the search engine did allow for the use of Boolean operators a complete or modified search syntax was used (Table S2). Total: Sum of records after removal of identified duplicates. (DOC) [file pntd.0002399.s005.doc]

**Table S1**

Searched bibliographic databases. Search syntax depended on whether or not the search engine did allow for the use of Boolean operators a complete or modified search syntax was used (supporting Table S2). Total: Sum of records after removal of identified duplicates.

| Database | URL | No. articles | Search syntax | Search date | Region | Comments |
| --- | --- | --- | --- | --- | --- | --- |
| PubMed/MEDLINE | http://www.ncbi.nlm.nih.gov/pubmed/ | 7767 | complete | 21/04/2010 | International |  |
| ISI Web of Knowledge | http://isiwebofknowledge.com/ | 8324 | complete | 22/04/2010 | International |  |
| Popline | http://www.popline.org/ | 70 | complete | 21/04/2010 | International |  |
| CAB Abstracts and Global Health | http://www.cabdirect.org/ | 27 | complete | 06/05/2010 | International |  |
| ProMed | http://www.promedmail.org | 241 | modified | 28/04/2010 | International |  |
| The Cochrane Library | http://www.thecochranelibrary.com | 11 | complete | 21/04/2010 | International |  |
| BIOLINE | http://www.bioline.org.br | 32 | modified | 29/04/2010 | International |  |
| WHOLIS | http://www.bireme.br | 17 | complete | 06/05/2010 | International |  |
| Health Information Locator | http://www.bireme.br | 3 | complete | 06/05/2010 | International |  |
| Institute of Tropical Medicine, Antwerp, Belgium | http://lib.itg.be:8000/webspirs/start.ws | 14 | complete | 05/05/2010 | International |  |
| King's Fund Information & Library Service | http://www.kingsfund.org.uk/library/http://kingsfund.koha-ptfs.eu/ | 0 | modified | 21/04/2010 | International | Grey literature |
| African Journals Online | http://ajol.info/ | 691 | modified | 05/05/2010 | Africa |  |
| African Index Medicus | http://indexmedicus.afro.who.int/ | 0 | modified | 21/04/2010 | Africa |  |
| Afro Library | http://afrolib.afro.who.int/ | 0 | modified | 21/04/2010 | Africa |  |
| Latin American and Caribbean Health Science | http://www.bireme.br | 496 | complete | 29/04/2010 | Latin America |  |
| MedCarib | http://www.bireme.br | 24 | complete | 06/05/2010 | Caribbean |  |
| REPIDISCA | http://www.bireme.br | 1 | complete | 06/05/2010 | Latin America and Caribbean |  |
| PAHO | http://www.bireme.br | 35 | complete | 06/05/2010 | Pan-America |  |
| IBECS | http://www.bireme.br | 94 | complete | 06/05/2010 | Spanish literature |  |
| CUIDEN | http://www.index-f.com/ | 2 | modified | 05/05/2010 | Spanish literature |  |
| HELLIS | http://www.hellis.org/ | 0 | modified | 21/04/2010 | Asia |  |
| Index Medicus for the South-East Asia Region | http://www.who.int/library/databases/searo/en/index.html | 82 | modified | 29/04/2010 | South-East Asia |  |
| Western Pacific Region Index Medicus | http://www.who.int/library/databases/wpro/en/index.html | 71 | modified | 29/04/2010 | Western Pacific |  |
| Indian Medlars Center - IndMed | http://indmed.nic.in/ | 22 | modified | 03/05/2010 | Indian literature |  |
| KoreaMed | http://www.koreamed.org/SearchBasic.php | 79 | modified | 03/05/2010 | Korean literature |  |
| Japan Science and Technology Information Aggregator | http://www.jstage.jst.go.jp/browse/ | 309 | modified | 05/05/2010 | Japanese literature |  |
| Health Research and Development Information Network | http://www.herdin.ph/ | 4 | modified | 03/05/2010 | Philippine literature |  |
| Index Medicus for the Eastern Mediterranean Region | http://www.who.int/library/databases/emro/en/index.html | 8 | modified | 28/04/2010 | Eastern Mediterranean |  |
| Panteleimon | www.panteleimon.org/maine.php3 | 8 | modified | 03/05/2010 | Russian literature |  |
| l'Ecole Nationale de la Santé Publique | http://www.bdsp.ehesp.fr/Base/ | 48 | complete | 05/05/2010 | French literature | Additional search in French |
| La Bibliothèque de Santé Tropicale | http://www.santetropicale.com/resume/catalogue.asp | 1 | modified | 05/05/2010 | Tropics, French literature | Additional search in French |
| System for Information on Grey Literature in Europe | opensigle.inist.fr | 4 | complete | 06/05/2010 | Europe | Grey literature |
| **Total** | **N/A** | **12176** | **N/A** | **N/A** | **N/A** |  |
