# Supplementary material for: Differences in Primary Sites of Infection between Zoonotic and Human Tuberculosis: Results from a Worldwide Systematic Review
Source: PLoS Negl Trop Dis. 2013 Aug 29;7(8):e2399. doi: 10.1371/journal.pntd.0002399 (PMC3757065; doi:10.1371/journal.pntd.0002399)
Supplement: Table S2 — Search syntax. Potentially relevant reports were identified using the search tools of the respective bibliographic databases (Table S1). At least one of the search terms under A had to be present in connection with at least one of the terms under B. The most sensitive search settings had been applied. If the respective search tool did not allow for the use of Boolean operators, all reports that were retrieved for any of the search terms under A were used. Search terms were translated into French for searches in French literature databases (Table S1). (DOC) [file pntd.0002399.s006.doc]

**Table S2**

Search syntax. Potentially relevant reports were identified using the search tools of the respective bibliographic databases (supporting Table S1). At least one of the search terms under A had to be present in connection with at least one of the terms under B. The most sensitive search settings had been applied. If the respective search tool did not allow for the use of Boolean operators, all reports that were retrieved for any of the search terms under A were used. Search terms were translated into French for searches in French literature databases (supporting Table S1).

| A: | B: |
| --- | --- |
| "bovine tuberculosis" | "zoonotic" |
| "bovine TB" | "zoonosis" |
| "BTB" | "patient" |
| "zoonotic tuberculosis" | "patients" |
| "zoonotic TB" | "cohort" |
| "animal tuberculosis" | "population" |
| "animal TB" | "person" |
| "Mycobacterium bovis" | "persons" |
| "M. bovis" | "people" |
| "scrofula" | "child" |
| "Pott's disease" | "children" |
| "lupus vulgaris" | "adult" |
|  | "adults" |
|  | "woman" |
|  | "women" |
|  | "man" |
|  | "men" |
|  | "human" |
|  | "humans" |
