# Supplementary material for: Differences in Primary Sites of Infection between Zoonotic and Human Tuberculosis: Results from a Worldwide Systematic Review
Source: PLoS Negl Trop Dis. 2013 Aug 29;7(8):e2399. doi: 10.1371/journal.pntd.0002399 (PMC3757065; doi:10.1371/journal.pntd.0002399)
Supplement: Table S3 — List of variables for data extraction of the eligible reports. (DOC) [file pntd.0002399.s007.doc]

**Table S3**

List of variables for data extraction of the eligible reports.

| **Variable** | **Description** |
| --- | --- |
| Data point | Unique identifier for each data point (data points have not been numbered, consecutively). |
| Record ID | Record ID as assigned during the screening for eligible records. More bibliographic information on the respective records can be found in table S3. The extracted data originates from these records or from studies cited within these records. In the latter case, the referenced paper is indicated 'Notes'. |
| Study | Author, journal and publication year of record relating to 'Record ID'. |
| WHO Region | Study region as defined by WHO (http://www.who.int/about/regions/en/index.html). |
| Geographical range | National (country-wide representative) or subnational (non-representative) data. |
| Setting | Detailed description of survey/study area. |
| Population coverge | Initial description of survey population (population tested for zoonotic TB). |
| Study population | Detailed description of survey population (population tested for zoonotic TB) |
| Sampling strategy | Sampling strategy. Notably, national or subnational surveillance generally covered the overall population of a given setting and reported all notified cases. |
| Case finding strategy | Active or passive case finding. |
| Detection method | Detection method applied to identify the causative agent of the alleged zoonotic TB case. |
| Causative agent | Causative agent of the alleged zoonotic TB case. |
| Changes diagnosis | Was the detection of cases standardized or were differences over time or between settings reported or probable? |
| Missing data | Was there a substantial amount of missing data affecting more than 10% of the sampled cases? |
| Age group | Age group or range of the survey population. |
| Gender | Gender of the survey population. |
| Study duration | (Estimated) study duration in months. The duration was calculated/estimated from the available information on the study period. |
| Study period | Study period as indicated in the survey report. |
| TB site 1 | Do examined patients of zoonotic TB suffer from pulmonary TB, extrapulmonary TB or TB affecting pulmonary and extrapulmonary? |
| TB site 2 | Site of extrapulmonary TB due to zoonotic TB as accurately as possible using the definitions used in the text or from ICD-10, Chapter I, blocks A15-A1 |
| Cases | Number of cases of alleged zoonotic TB detected among the survey population. |
| Tested | Number of individuals included in the survey population. |
| Note | Comments in relation to the data above. Note that if the data extracted originated from a survey referenced in a given report, this survey is referenced, here. |
