# Supplementary material for: Differences in Primary Sites of Infection between Zoonotic and Human Tuberculosis: Results from a Worldwide Systematic Review
Source: PLoS Negl Trop Dis. 2013 Aug 29;7(8):e2399. doi: 10.1371/journal.pntd.0002399 (PMC3757065; doi:10.1371/journal.pntd.0002399)
Supplement: Table S4 — Information on the included 27 records stratified by WHO region. (DOC) [file pntd.0002399.s008.doc]

**Table S4**

Information on the included 27 records stratified by WHO region.

| **ID** | **authors** | **title** | **Pub date** | **journal** | **country** | **Study period** | **Study size** | **Identi-fication method** | **used for^b^** |
| --- | --- | --- | --- | --- | --- | --- | --- | --- | --- |
| **Africa (WHO region 1)** | | | | |  | | | | |
| 140 | Rasolofo R;Quirin R;Rapaoliarijaona A;Rakotoaritahina H;Vololonirina EJ;Rasolonavalona T;Ferdinand S;Sola C;Rastogi N;Ramarokoto H;Chanteau S | Usefulness of restriction fragment length polymorphism and spoligotyping for epidemiological studies of Mycobacterium bovis in Madagascar: description of new genotypes | 2006 | Vet Microbiol | Madagascar | 1994- 2000 | 18 | mol | 1,2 |
| **America (WHO region 2)** | | | | |  | | | | |
| 485a^a^ | Grange JM;Yates MD | Zoonotic aspects of Mycobacterium bovis infection | 1994 | Vet Microbiol | Canada, Ontario | 1964 -1970 | 31 | n.a. | 1,2 |
| 317 | Dankner WM;Davis CE | Mycobacterium bovis as a significant cause of tuberculosis in children residing along the United States-Mexico border in the Baja California region | 2000 | Pediatrics | USA, California, San Diego County | 1980 -1997 | 61 | chem | 1,2, 3,4 |
| 164 | Lobue PA;Moser KS | Treatment of Mycobacterium bovis infected tuberculosis patients: San Diego County, California, United States, 1994-2003 | 2005 | Int J Tuberc Lung Dis | USA, California, San Diego County | 1994 - 2003 | 167 | chem | 1,3 |
| 51 | Wilkins MJ;Meyerson J;Bartlett PC;Spieldenner SL;Berry DE;Mosher LB;Kaneene JB;Robinson-Dunn B;Stobierski MG;Boulton ML | Human Mycobacterium bovis infection and bovine tuberculosis outbreak, Michigan, 1994-2007 | 2008 | Emerg Infect Dis | USA, Michigan | 1994 -2007 | 13 | mol | 1,2 |
| 46 | Hlavsa MC;Moonan PK;Cowan LS;Navin TR;Kammerer JS;Morlock GP;Crawford JT;Lobue PA | Human tuberculosis due to Mycobacterium bovis in the United States, 1995-2005 | 2008 | Clin Infect Dis | 41 States of the USA | 1995 -2005 | 165 | mol | 1,2, 3,4 |
| 39 | Cicero R;Olivera H;Hernandez-Solis A;Ramirez-Casanova E;Escobar-Gutierrez A | Frequency of Mycobacterium bovis as an etiologic agent in extrapulmonary tuberculosis in HIV-positive and -negative Mexican patients | 2009 | Eur J Clin Microbiol Infect Dis | Mexico, Hospital General in Mexico City | 2000 - 2003 | 17 | mol | 2,4 |
| 155; 1191- 1193 | anonymous | Human tuberculosis caused by Mycobacterium bovis--New York City, 2001-2004; PRO/AH/EDR> Mycobacterium bovis, cheese - USA (NY): alert (1-3) | 2005 | MMWR Morb Mortal Wkly Rep; http://www.promedmail.org/ | USA, New York City | 2001 -2004 | 35 | mol | 1,2 |
| 48 | Rodwell TC;Moore M;Moser KS;Brodine SK;Strathdee SA | Tuberculosis from Mycobacterium bovis in binational communities, United States | 2008 | Emerg Infect Dis | USA, California, San Diego County | 2001 -2005 | 132 | mol | 1,3 |
| **Europe (WHO region 4)** | | | | |  | | | | |
| 598 | Grange JM;Collins CH | Bovine tubercle bacilli and disease in animals and man | 1987 | Epidemiol Infect | England | 1927 | 264 | n.a. | 1,2, 3,4 |
| 338 | Robert J;Boulahbal F;Trystram D;Truffot-Pernot C;de Benoist AC;Vincent V;Jarlier V;Grosset J | A national survey of human Mycobacterium bovis infection in France. Network of Microbiology Laboratories in France | 1999 | Int J Tuberc Lung Dis | France | 1995 | 38 | chem | 1,2 |
| 485b^a^ | Grange JM;Yates MD | Zoonotic aspects of Mycobacterium bovis infection | 1994 | Vet Microbiol | Southern Sweden | 1936 -1939 | 94 | n.a. | 1,2 |
| 485c^a^ | Grange JM;Yates MD | Zoonotic aspects of Mycobacterium bovis infection | 1994 | Vet Microbiol | England and Wales | 1962 -1966 | 102 | n.a. | 1,2 |
| 612 | Wilkins EG;Griffiths RJ;Roberts C | Pulmonary tuberculosis due to Mycobacterium bovis | 1986 | Thorax | England, Merseyside region | 1969 -1984 | 77 | chem | 1,3 |
| 588 | Yates MD;Grange JM | Incidence and nature of human tuberculosis due to bovine tubercle bacilli in South-East England: 1977-1987 | 1988 | Epidemiol Infect | South-East England | 1977 -1987 | 201 | chem | 1,2 |
| 149 | Esteban J;Robles P;Soledad JM;Fernandez Guerrero ML | Pleuropulmonary infections caused by Mycobacterium bovis: a re-emerging disease | 2005 | Clin Microbiol Infect | Spain, University Hospital | 1980 - 2003 | 13 | mol | 1,2 |
| 425 | Cotter TP;O'Shaughnessy E;Sheehan S;Cryan B;Bredin CP | Human Mycobacterium bovis infection in the south-west of Ireland 1983-1992: a comparison with M. tuberculosis | 1996 | Ir Med J | South-West region of Ireland | 1983 -1992 | 30 | chem | 1,2, 3 |
| 515 | Sauret J;Jolis R;Ausina V;Castro E;Cornudella R  Hardie | Human tuberculosis due to Mycobacterium bovis: report of 10 cases | 1992 | Tuber Lung Dis | Spain, Barcelona, Sant Pau's Hospital | 1986 -1990 | 10 | chem | 1,2 |
| 521 | Hardie RM;Watson JM | Mycobacterium bovis in England and Wales: past, present and future | 1992 | Epidemiol Infect | England and Wales | 1986 -1990 | 228 | n.a. | 1,2 |
| 162 | Min SM;Kelly P;Byrne C;Clancy L | Antibiotic resistant tuberculosis and bovine tuberculosis in an Irish hospital population (1991 to 2001) | 2005 | Ir Med J | Ireland, Dublin, St. James's Hospital | 1991 -2001 | 19 | n.a. | 1 |
| 74 | Evans JT;Smith EG;Banerjee A;Smith RM;Dale J;Innes JA;Hunt D;Tweddell A;Wood A;Anderson C;Hewinson RG;Smith NH;Hawkey PM;Sonnenberg P | Cluster of human tuberculosis caused by Mycobacterium bovis: evidence for person-to-person transmission in the UK | 2007 | Lancet | United Kingdom | 1994 -2004 | 296 | mol | 1 |
| 219 | Kubica T;Rusch-Gerdes S;Niemann S | Mycobacterium bovis subsp. caprae caused one-third of human M. bovis-associated tuberculosis cases reported in Germany between 1999 and 2001 | 2003 | J Clin Microbiol | Germany | 1999 - 2001 | 166 | mol | 1 |
| 87 | Mignard S;Pichat C;Carret G | Mycobacterium bovis infection, Lyon, France | 2006 | Emerg Infect Dis | France, Lyon | 2000 -2005 | 11 | mol | 1,2 |
| 13 | Rodriguez E;Sanchez LP;Perez S;Herrera L;Jimenez MS;Samper S;Iglesias MJ | Human tuberculosis due to Mycobacterium bovis and M. caprae in Spain, 2004-2007 | 2009 | Int J Tuberc Lung Dis | 11 regions in Spain | 2004 - 2007 | 110 | mol | 1 |
| **Western Pacific (WHO region 6)** | | | | |  | | | | |
| 337 | Cousins DV;Dawson DJ | Tuberculosis due to Mycobacterium bovis in the Australian population: cases recorded during 1970-1994 | 1999 | Int J Tuberc Lung Dis | Australia | 1970 -1994 | 148 | chem | 1,2 |
| 134 | Baker MG;Lopez LD;Cannon MC;de Lisle GW;Collins DM | Continuing Mycobacterium bovis transmission from animals to humans in New Zealand | 2006 | Epidemiol Infect | New Zealand | 1998 -2002 | 34 | mol | 1,3 |
| 55 | Jou R;Huang WL;Chiang CY | Human tuberculosis caused by Mycobacterium bovis, Taiwan | 2008 | Emerg Infect Dis | China, Taiwan | 2004 -2005 | 15 | mol | 1,2 |

^a^as ID 485 reported on three different regions it is listed three times

^b^further used for (number of reports):

1: proportion of extrapulmonary TB site (26)

2: proportion of specific site among extrapulmonary TB (19)

3: direct comparison of *M. bovis* and *M. tuberculosis* cases for proportion of extrapulmonary TB site (8)

4: direct comparison of *M. bovis* and *M. tuberculosis* cases for specific site among extrapulmonary TB (4)
